# Tobacco smoking and associated factors in human immunodeficiency virus-infected adults attending human immunodeficiency virus clinics in the Western Cape province, South Africa

**DOI:** 10.4102/sajhivmed.v21i1.1072

**Published:** 2020-04-21

**Authors:** Muyunda Mutemwa, Nasheeta Peer, Anniza de Villiers, Mieke Faber, Andre-Pascal Kengne

**Affiliations:** 1 Non-Communicable Diseases Research Unit, South African Medical Research Council, Cape Town, South Africa; 2 Department of Medicine, University of Cape Town, Cape Town, South Africa; 3 Research Capacity Development Division, South African Medical Research Council, Cape Town, South Africa

**Keywords:** HIV and AIDS, smoking, cotinine, prevalence, South Africa

## Abstract

**Background:**

In human immunodeficiency virus (HIV)-infected individuals, smoking increases both HIV-related and non-related negative health outcomes.

**Objectives:**

To determine the prevalence and associations of smoking in HIV-infected adults receiving antiretroviral therapy at public healthcare facilities in the Western Cape province, South Africa.

**Methods:**

Participants comprised 827 HIV-infected patients, who were > 18 years old and randomly selected from 17 HIV healthcare facilities. Self-reported smoking was defined as smoking tobacco daily or occasionally. Serum cotinine levels confirmed smoking status.

**Results:**

Participants included 653 women and 174 men. The overall mean (standard deviation [SD]) age was 38.9 (9.0) years, 41.1 (8.9) years in men and 37.7 (8.9) years in women (*p* ˂ 0.001). The median diagnosed duration of HIV infection was 5 years. Smoking prevalence was 22% overall, and 26% in men and 21% in women (*p* = 0.022). The prevalence of former smoking was 14%. About a quarter of participants (185/751; 24.6%) had serum cotinine levels > 100 mg/mL with similar prevalence of high levels across smoking status (current smokers: 27.2%, former smokers: 29.6% and never smokers: 22.7%, *p* = 0.564) and did not vary by age, gender, cluster of differentiation 4 count or known duration of HIV. There was no agreement between self-reports and cotinine levels at ranking smoking exposure.

**Conclusions:**

Prevalence of current tobacco smoking in HIV-infected patients on care is within the range of that in the general population. This highlights the potential missed opportunity or challenges of co-addressing smoking cessation in individuals already in regular contact with the health system.

## Introduction

Globally, over 1.1 billion men and women ≥ 15 years of age currently smoke tobacco. Consequently, tobacco use is a growing public health burden worldwide that was responsible for 7.1 million deaths in 2016.^1^ Even in South Africa (SA), despite the introduction of comprehensive legislative action to discourage tobacco use since the early 1990s, tobacco smoking remains a major public health problem.^2^ In 2016, 37% of men and 8.0% of women ≥ 15 years of age smoked tobacco.^3^ Tobacco smoking contributes to a large burden of the preventable disease accounting for 8.0% – 9.0% of mortality in SA.^2^ While lung cancer had the largest attributable fraction because of tobacco smoking, cardiovascular diseases contributed to the largest proportion of deaths caused by smoking.

The impact of smoking extends beyond the well-known consequences of tobacco use to other conditions including human immunodeficiency virus (HIV) infection. In people living with HIV or acquired immunodeficiency syndrome (AIDS) (PLWHA), the harmful effects of smoking are greater and threaten efforts in controlling HIV and AIDS.^1^ Smoking increases both HIV-related and non-related outcomes and has been shown to impact HIV disease progression.^4,5^ Nevertheless, smoking prevalence in PLWHA is approximately twofold to threefold higher than in the general population in developed countries and ranges from 40% to 74%.^6,7^

The greater adverse effects of tobacco smoking in PLWHA are highly relevant to SA. The country has the greatest burden of HIV worldwide with approximately 7.97 million PLWHA in 2019.^8^ With almost 20% of 15–49-year-old South African adults being HIV positive, determining the burden of tobacco smoking in the HIV infected can inform strategies for tobacco cessation in this high-risk population. This is particularly pertinent in the era of widespread dissemination of antiretroviral therapy (ART) in SA^9^ and increased longevity of the HIV-infected population. People living with HIV and AIDS are now at an increased risk of dying from cardiovascular and other non-communicable diseases (NCDs), including tobacco-related conditions, rather than from AIDS.^10,11,12^

This study, therefore, aims to determine the prevalence of smoking and associated factors including HIV-specific factors in PLWHA receiving ART at public healthcare facilities in the Western Cape province of SA.

## Methods

### Population and sampling

This cross-sectional study was conducted in a sample of ≥ 18-year-old HIV-infected adults who were randomly selected from a list of patients attending the clinic on the study day. Participants were recruited between March 2014 and February 2015 from healthcare facilities in the Western Cape that provided ART to at least 325 HIV-infected patients per month. This was to ensure the recruitment of an adequate number of participants within a reasonable period. Of the 17 healthcare facilities selected, 10 were in Cape Town and seven were in the surrounding rural municipalities. Excluded participants were those who were pregnant, breastfeeding, bedridden, undergoing cancer treatment, on corticosteroid treatment, or unwilling or unable to provide consent. The detailed methods have been described previously.^13^

### Data collection

Trained clinicians, nurses and fieldworkers collected data via standardised international questionnaires, clinical measurements and biochemical analyses. Data were captured on personal digital assistants (PDAs), using electronic case report forms with built-in checks for quality control. The interviews and physical examinations were conducted on the recruitment day, and following an overnight fast, participants returned the next day to have their blood samples taken.

Participants provided their socio-demographic history, including tobacco use, which was adapted from the World Health Organization’s (WHO) STEPwise approach to Surveillance (STEPS) tool.^14^ Information on the duration of diagnosed HIV infection, cluster of differentiation 4 (CD4) counts and ART regimens was extracted from clinical records. Height to the nearest millimetre was measured using a Leicester Height Scale (Seca, UK) with the participant barefoot and in the upright position. Weight to the nearest gram was measured using Analog and Digital (A&D) Medical PersonalScale (Model UC-321, Japan) with the participant in light clothes and without shoes. Biochemical analyses included serum cotinine levels and lipid profiles, which were acquired at an ISO 15189-accredited pathology laboratory (Path Care, Reference Laboratory, Cape Town, SA), as previously described in detail.^15^

### Definitions

Participants were classified as either ‘current smoker’, ‘former smoker’ or ‘never a smoker’, considering all forms of smoked tobacco, including cigarettes, cigars or pipes. Current smokers included participants who smoked daily or occasionally. Former smokers refer to participants who indicated that they had quit smoking at the time of the interview, regardless of the duration since quitting. ‘Smokeless tobacco users’ referred to the use of chewing tobacco, snuff or betel leaf and the areca nut at the time of the survey. Exposure to second-hand smoke was determined from ‘household smoke’. Cotinine, a major metabolite of nicotine, is commonly used as a biomarker to identify exposure to tobacco.^16,17^ Serum cotinine levels were used to define the different smoking categories as follows: ‘no tobacco exposure’: cotinine <10 mg/mL, ‘environmental smoke exposure or light smoking’: cotinine levels of 10 ng/mL – 100 ng/mL and ‘moderate to heavy smoking’: cotinine >100 mg/mL, in line with the 2012 South African National Health and Nutrition Examination Survey (SANHANES).^18^

Alcohol use was defined as drinking at least one standard alcoholic drink per day. A standard alcoholic drink consists of a can (340 mL) of beer, one glass (125 mL) wine or ‘one-shot’ (25 mL) of spirits. Body mass index (BMI) was calculated as weight in kilograms divided by height in metres squared (kg/m^2^), and overweight and obesity was defined as BMI ≥ 25 kg/m^2^.^19^ Cut-points for HIV-related variables were set at median values, that is, ≥ 396 cell/mm^3^ for CD4^+^ counts and of ≥ 5 years duration of HIV diagnosis.

### Statistical analysis

The Statistical Package for Social Sciences (IBM SPSS Inc, Chicago, IL, USA) V.25.0 software was used for the data analyses. Continuous variables are presented as means (± standard deviation [SD]) or medians (25th – 75th percentiles) and categorical variables are presented as counts and percentages. Analysis of variance (ANOVA), *χ*^2^ tests and non-parametric equivalents were used as appropriate for group comparisons. Logistic regression models adjusted for age and gender were used to determine associations with current smoking. A *p*-value < 0.05 defined statistically significant results.

### Approval to conduct the study

Permission to conduct the survey was obtained from the Health Research Office of the Western Cape Department of Health and the relevant healthcare facilities. The study was approved by the South African Medical Research Council Ethics Committee and conducted in accordance with the principles of the Declaration of Helsinki. All participants signed informed consent. Data were anonymised to prevent identification of individual participants.

### Ethical consideration

Permission to conduct the survey was obtained from the Health Research Office of the Western Cape Department of Health and the relevant healthcare facilities. The study was approved by the South African Medical Research Council Ethics Committee (reference number: EC021-11/2013) and conducted in accordance with the principles of the Declaration of Helsinki. All participants signed informed consent.

## Results

The sample for this analysis comprised 827 participants, 653 (79%) women and 174 (21%) men after the exclusion of four with missing information on smoking status. The mean age was 38.4 years overall, with men significantly older than women (41.1 vs. 37.7 years, *p* < 0.001) (Table 1). Compared to men, women had fewer years of education, but a higher proportion was employed. Men were more likely to consume alcohol than women (54.0% vs. 34.3%, *p* < 0.001). Compared with men, women were more likely to have higher BMI and prevalence of overweight and obesity (both *p* < 0.001). Median duration of diagnosed HIV infection (*p* = 0.048) and median CD4 counts (*p* = 0.001) were also higher in women compared with men. The lipid profile did not vary by gender.

**TABLE 1:** Characteristics of human immunodeficiency virus patients presented by gender and smoking status.

Variables	Male patients (*N* = 174; 21%)		Female patients (*N* = 653; 79%)		*p*	Current smoker (*N* = 181; 22%)		Former smoker (*N* = 118; 14%)		Never smoker (*N* = 528; 64%)		*p*
	*n*	%	*n*	%		*n*	%	*n*	%	*n*	%	
Age in years, mean (SD)	41.1	8.9	37.7	8.9	< 0.001	39.3	10.1	38.3	8.6	38.1	8.7	0.332
Age group in years												
≤ 34	64	36.8	242	37.1	0.167	71	39.2	45	38.1	190	36.0	0.649
35–44	68	39.1	257	39.4	-	69	38.1	41	34.7	215	40.7	-
45–54	28	16.1	127	19.4	-	29	16.0	26	22.0	100	18.9	-
≥ 55	14	8.0	27	4.1	-	12	6.6	6	5.1	23	4.4	-
Education level												
≤ Grade 12	161	92.5	633	96.9	0.008	176	97.2	114	96.6	504	95.5	0.536
> Grade 12	13	7.5	20	3.1	-	5	2.8	4	3.4	24	4.5	-
Occupation												
Employed	91	52.3	373	57.1	0.031	94	51.9	68	57.6	302	57.2	0.345
Unemployed	46	26.4	109	16.7	-	42	23.2	26	22.0	87	16.5	-
Pensioners	7	4.0	35	5.4	-	10	5.5	6	5.1	26	4.9	-
Other	30	17.2	136	20.8	-	35	19.3	18	15.3	113	21.4	-
Marital status												
Married	37	21.3	119	18.2	0.516	39	21.5	21	17.8	96	18.2	0.305
Never married	113	64.9	430	65.8	-	108	59.7	83	70.3	352	66.7	-
Divorced or separated	19	10.9	70	10.7	-	21	11.6	12	10.2	56	10.6	-
Widowed	5	2.9	34	5.2	-	13	7.2	2	1.7	24	4.5	-
Use of smokeless tobacco products	4	2.3	14	2.1	0.901	1	0.6	3	2.5	14	2.7	0.237
Second-hand smoke	78	44.8	384	58.8	0.001	103	56.9	59	50.0	300	56.8	0.383
Serum cotinine (*N* = 751)	-	-	-	-	-	-	-	-	-	-	-	0.564
< 10 ng/mL	91/152	59.96	367/599	61.3	0.940	95/158	60.1	63/108	58.3	300/485	61.9	-
10 ng/mL – 100 ng/mL	23/152	15.1	85/599	14.2	-	18/158	11.4	17/108	15.7	73/485	15.1	-
> 100 ng/mL	38/152	25.0	147/599	24.5	-	45/158	28.5	28/108	25.9	112/485	23.1	-
Alcohol use	94	54.0	224	34.3	< 0.001	76	42.0	45	38.1	197	37.3	0.535
LDL-C, mmol/L, median (25th – 75th percentiles)	2.5	2.0–3.1	2.5	2.1-3.1	0.684	2.5	2.1-3.1	2.4	2.0-2.8	2.5	2.1-3.1	0.492
HDL-C, mmol/L, median (25th – 75th percentiles)	1.2	1.0–1.4	1.2	1.0-1.5	0.925	1.2	1.0-1.5	1.3	1.1-1.5	1.2	1.0-1.5	0.888
Triglycerides mmol/L, median (25th – 75th percentiles)	1.0	0.7– 1.3	1.0	0.7-1.4	0.156	1.0	0.7-1.4	1.0	0.7-1.3	1.0	0.8-1.4	0.476
Total Cholesterol, mmol/L, median (25th – 75th percentiles)	4.2	3.7–4.8	4.4	3.8-5.0	0.298	4.5	3.8-5.0	4.2	3.9-4.7	4.4	3.7-5.1	0.700
BMI in kg/m^2^, median (25th – 75th percentiles)	21.2	20.1-23.4	28.8	23.3-33.8	< 0.001	25.5	21.4-31.8	28.3	22.3-34.7	27.9	22.8-32.8	0.504
Overweight or obese	47	27.0	474	72.6	< 0.001	106	58.6	70	59.3	345	65.3	0.178
Duration in years, median (25th – 75th percentiles)	4	2–8	5	2-9	0.048	6	3-10	4	2-6	5	2-9	0.020
< Median of 5 years	80/159	50.3	274/606	45.2	0.254	81/168	48.2	61/108	56.5	212/489	43.4	0.040
≥ Median of 5 years	79/159	49.7	332/606	54.8	-	87/168	51.8	47/108	43.5	277	56.6	-
CD4+ count, median (25th – 75th percentiles) (*N* = 388)	272	168–460	413	248-616	0.001	405	265-564	442	239-706	373	218-604	0.528
< Median of 396 cells/mm^3^	39/55	70.9	155/333	46.5	0.001	43/85	50.6	20/53	37.7	131/250	52.4	0.151
≥ Median of 396 cells/mm^3^	16/55	29.1	178/333	53.5	-	42/85	49.4	33/53	62.3	191/250	47.6	-

Note: Alcohol use was recorded as consumption of at least one standard alcoholic drink per day.

LDL-C, low-density lipoprotein cholesterol; HDL-C, high-density lipoprotein cholesterol; BMI, body mass index; CD4, cluster of differentiation 4; HIV, human immunodeficiency virus; SD, standard deviation.

**FIGURE 1:**
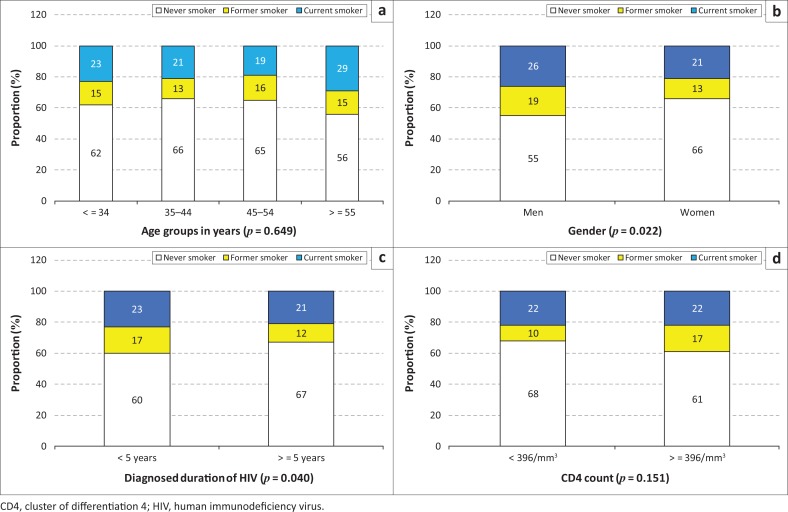
Smoking status in different subgroups defined by (a) age, (b) gender, (c) known duration of human immunodeficiency virus infection and (d) cluster of differentiation 4 count.

Current smoking prevalence was 22% overall and 26% in men and 21% in women (*p* = 0.022) (Table 1 and Figure 1). Overall, 14% of the participants were former smokers (Table 1). There were no significant trends in smoking status by age category (*p* = 0.649). Smoking status was also marginally related to the known duration of HIV infection (*p* = 0.040) but not the CD4 count (*p* = 0.151) (Figure 1).

Exposure to second-hand smoke was high with significantly higher rates in women (58.8%) compared with men (44.8%) (*p* = 0.001) (Table 1). The use of smokeless tobacco products was low, at < 3% for any sub-category, with no significant differences by gender or smoking status.

The median duration of diagnosed HIV infection was 6 years in current smokers, 5 years in non-smokers and 4 years in former smokers (*p* = 0.020). There were no significant differences in the median CD4 counts by smoking status. In age- and gender-adjusted logistic regression models, none of the general and HIV-predictive characteristics was associated with current smoking (Table 2).

**TABLE 2:** Logistic regression for the associations with current smoking in human immunodeficiency virus-infected patients.

Variables	Adjusted OR	*p*
Age	0.99 [0.97–1.01]	0.200
Gender	-	0.229
Male patients	1.00	-
Female patients	1.27 [0.86–1.89]	-
Education	-	-
≤Grade 12	1.00	-
>Grade 12	1.30 [0.87–1.93]	0.298
Employment status	-	0.452
Employed	1.00	-
Unemployed	0.72 [0.47–1.10]	-
Pensioners	0.87 [0.41–1.85]	-
Other	1.02 [0.65–1.61]	-
Marital status	-	0.139
Married	1.00	-
Never married	1.33 [0.87–1.96]	-
Divorced or separated	1.07 [0.58–1.96]	-
Widowed	0.63 [0.30–1.36]	-
Smokeless tobacco smoking	5.46 [0.72–41.73]	0.102
Household tobacco	0.93 [0.66–1.30]	0.675
Alcohol use	0.84 [0.60–1.19]	0.331
BMI in kg/m^2^	-	0.299
Normal BMI ˂ 25	1.00	-
Overweight or obese ≥ 25	0.21 [0.84–1.76]	-
Current tuberculosis	1.07 [0.77–1.50]	0.685
Duration of HIV+ diagnosis	-	0.627
< Median of 5 years	1.00	-
≥ Median of 5 years	1.09 [0.77–1.54]	-
CD4+ count in cells/mm^3^	-	0.954
≥ Median of 396 cells/mm^3^	1.00	-
< Median of 396 cells/mm^3^	0.98 [0.60–1.62]	-

Note: Alcohol use was recorded as consumption of at least one standard alcoholic drink per day.

BMI, body mass index; CD4, cluster of differentiation 4; HIV, human immunodeficiency virus; OR, odds ratio.

**FIGURE 2:**
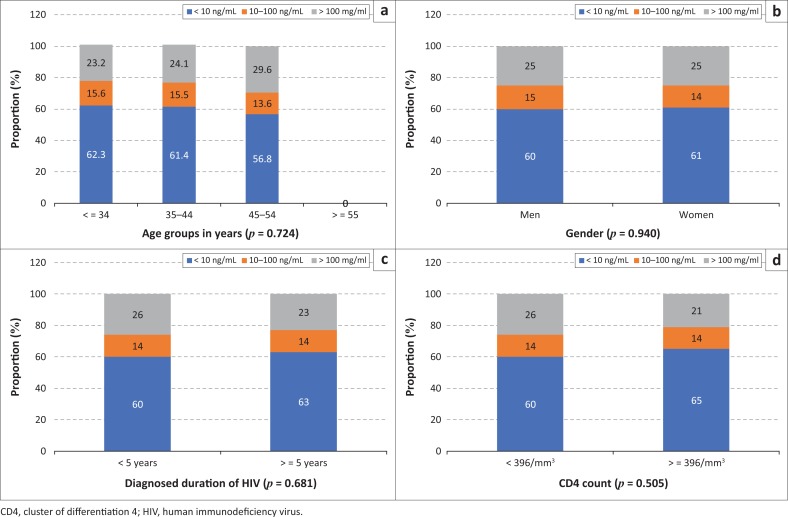
Distribution of cotinine strata by (a) age group, (b) gender, (c) known duration of human immunodeficiency virus infection and (d) cluster of differentiation 4 count.

Data on serum cotinine were available for 751 participants. About a quarter of these participants had serum cotinine levels > 100 mg/mL, indicating exposure to tobacco smoke. Prevalence of high serum cotinine levels was similar across smoking status (current smokers: 28.5%, former smokers: 25.9% and never smokers: 23.1%) (*p* = 0.564) and did not differ between men and women (*p* = 0.940), between those above and below median duration of diagnosed HIV infection (*p* = 0.681) and between those above and below median CD4 count (*p* = 0.505) (Figure 2).

**FIGURE 3:**
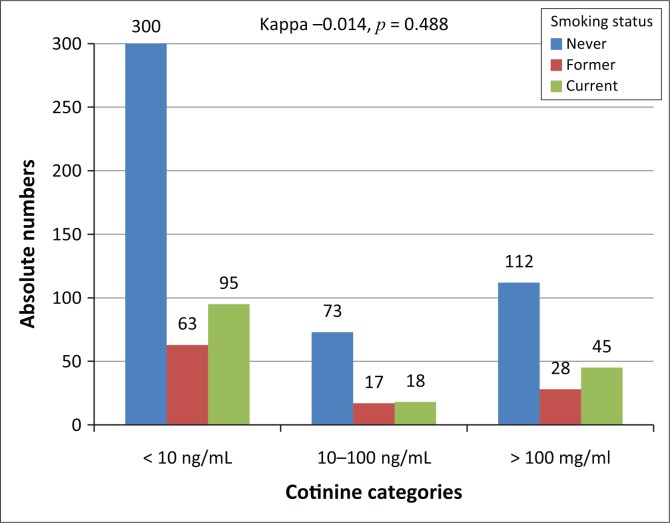
Agreement between self-reports and cotinine levels at ranking smoking status.

Figure 3 shows the cross-classification of smoking exposure by self-reports and serum cotinine levels, revealing the lack of agreement between the two classification methods (kappa = -0.014, *p* = 0.488). Among the 751 participants with data available on serum cotinine levels, 158 (21.0%) were current smokers based on self-reports, 108 (14.4%) were former smokers, and 485 (64.6%) had never smoked. Among current smokers based on self-reports, 95 (60.1%) had cotinine levels lower than 10 ng/mL, indicative of low tobacco exposure, while 45 (28.5%) had cotinine levels above 100 ng/mL, indicative of moderate-to-heavy smoking. Among those who had never smoked, 300 (61.9%) had no exposure to tobacco based on cotinine levels, while 112 (23.1%) had cotinine levels compatible with moderate-to-heavy smoking (Figure 3).

## Discussion

Our data show that over one in five PLWHA currently smoke tobacco, with men being more likely to do so than women and with no indication that smoking habits were influenced either by the duration or by the time since HIV diagnosis and awareness of the nadir CD4 count. Furthermore, over half of the study samples (including those who had never smoked) were exposed to second-hand smoke, with such exposure being higher in women. For participants with data available on serum cotinine concentrations, about a quarter (including among self-declared never-smokers) had cotinine concentrations indicative of moderate-to-heavy tobacco smoke exposure. Altogether, our findings suggest that despite the frequent contact of PLWHA with the health system, multiple opportunities had been missed to address the harmful effects of smoking or implement smoking cessation programmes.

Current estimates of smoking habits in the South African population are, in general, based on the 2012 SANHANES^18^ and the 2016 South African Demographic Health Survey (SADHS).^3^ According to the SANHANES, 20.8% (32.8% in men and 10.1% in women) of the general population ever smoked (which include current and former smokers) with 38.5% in the Western Cape province. Furthermore, about two-thirds of participants had detectable cotinine in the blood, suggesting recent exposure to cigarette smoking. In the 2016 SADHS, 37% of men and 8% of women aged 15 years and above reported currently smoking tobacco products regularly or occasionally. Equivalent figures for the Western Cape province were 43% and 26%. The prevalence of smoking in our sample, therefore, seems to be generally in line with recent estimates in the general population at the national level.

Few other studies reported smoking habits among PLWHA in SA. In a sample of 1210 PLWHA in Klerksdorp, Elf and co-workers^20^ found a 34% prevalence of ever-smokers, with rates being higher in men than in women, in line with our findings. In an earlier study in a much smaller sample, Waweru and co-workers reported prevalence rates of 15% (men vs. women: 23.2% vs. 7.4%) for current smoking in Johannesburg.^21^ Studies from other African countries suggest rates of smoking in PLWHA lower than those reported in SA; likely reflecting the relatively lower overall prevalence of smoking in the general population in these countries.^22,23^ In an analysis of Demographic Health Survey data from 27 low- and middle-income countries including 24 African countries (excluding SA), the overall prevalence of tobacco smoking in PLWHA across African countries was 24.2% in men (ranging from 9.7% in Ethiopia to 54.8% in The Gambia) and 1.0% in women (ranging from 0% in 11 countries to 4.4% in Gabon).^24^ Across these surveys, the risk ratio (RR) comparing the prevalence of smoking in people with versus without HIV was in favour of a 47% (male) and 87% (female) relatively higher prevalence in PLWHA. In about half of the studies, however, the confidence interval around RR generally crossed the unity, indicating no significant difference.^24^ The male preponderance in smoking uptake in the general population has largely been described. This gender difference extends to PLWHA in some studies but narrows down (as in ours) or even disappears completely in some, suggesting an increased uptake of the habit in women.^25^

One observation from our study was the lack of agreement between self-reports and measured cotinine at ranking status for smoking exposure. Assuming that bias in self-reported status would tend to favour concealing current smoking as opposed to wrongly claiming such a status, applying cotinine levels selectively only in former or never smokers, would have identified nearly an additional 20% of the total samples who were likely current smokers. This would nearly double the proportion of current smokers, suggesting that the dependence on self-report alone is likely to underestimate the true magnitude of current smoking among PLWHA in care. This assumption, however, must be considered in the context of the validity of the cotinine cut-offs applied in our study.

The harmful effects of smoking in PLWHA have been largely described.^26^ Smoking-related health hazards seen in the general population are exacerbated in PLWHA, where smoking is also responsible for some harmful health effects that are specific to this vulnerable population. People living with HIV and AIDS who smoke are at high risk of cancers (including non-AIDS defining cancers), chronic obstructive pulmonary diseases (COPDs) and chest infections. There are suggestions that smoking can also limit the benefits of ART and decrease life expectancy even in the context of adequate viral suppression^27^; nevertheless, achieving smoking cessation in PLWHA is likely more challenging than in the general population. Successful and sustainable smoking strategies are therefore needed to mitigate the risk of adverse health outcomes in PLWHA.

Given the burden of cigarette smoking and its adverse health outcomes among HIV-positive patients, screening for smoking and support to quit should be integrated into HIV and AIDS treatment programmes. Currently, evidence exists for both pharmacological and non-pharmacological interventions for smoking cessation, but evidence is needed on how they can best be implemented for smoking cessation in PLWHA in African countries.^26,28^ One recent qualitative review of smoking cessation interventions in PLWHA identified 32 publications reporting on 28 interventions.^29^ These studies essentially originated from western countries and the USA in particular. Thirteen of the interventions tested resulted in improved smoking cessation outcomes, with information and communication technologies and clinic-based interventions having the greatest potential to achieve smoking cessation among PLWHA. This is a significant observation considering that with regard to HIV care, PLWHA constitute a highly medicalised population, and are familiar with mHealth interventions in HIV care and monitoring.^30^ Another recent comparative meta-analysis concluded that compared with face-to-face, interventions mHealth interventions could better achieve smoking cessation in the short term in PLWHA.^31^ Besides the inadequate knowledge on the efficacy of interventions to achieve smoking cessation in PLWHA, other identified barriers hampering smoking cessation interventions in PLWHA include the scepticism of healthcare providers regarding certain interventions such as nicotine replacement, their unpreparedness to co-address smoking cessation during routine HIV care and other competing priorities.^26^ In the specific case of SA, economic, social or interpersonal and individual-level factors including stress have been suggested as barriers hindering smoking cessation in PLWHA.^32^

### Strengths and limitations

This study has some limitations. Participants were recruited from only one province of SA and included predominantly women. Smoking assessment inconsistently collected data on the age at initiation (or cessation) of smoking, limiting our ability to assess the potential effect of HIV diagnosis on the adoption or cessation of smoking habits. Data were missing on HIV characteristics (CD4 count) in an important number of participants, limiting our statistical power for some sub-group analyses. The study included only PLWHA and therefore did not offer the opportunity of comparing estimates with those in the non-HIV-infected population. Our study also has some strengths including the relatively large and randomly selected sample, which increased the generalisability of our findings. We also had data on blood cotinine in about 90% of our sample, which allowed us to substantiate that self-reports alone likely misclassify smoking status, with nearly a quarter of those reporting never smoking, having blood cotinine levels compatible with current moderate-to-heavy smoking.

## Conclusion

People living with HIV and AIDS in care have current tobacco smoking rates within the range of those found in the general population. These rates appear similar regardless of the known duration of HIV infection and status of disease control. This highlights the potential of missed opportunities or the challenges of co-addressing smoking cessation in PLWHA who are already in regular contact with the health system for the management of HIV and related co-morbidities. With the improved survival of PLWHA on ART and the emergence of NCDs as a new threat to the health of this population, proactively addressing smoking and other major NCD risk factors must become an integral part of the routine care of PLWHA.
